# Acceleration of short and long DNA read mapping without loss of accuracy using suffix array

**DOI:** 10.1093/bioinformatics/btu553

**Published:** 2014-08-20

**Authors:** Joaquín Tárraga, Vicente Arnau, Héctor Martínez, Raul Moreno, Diego Cazorla, José Salavert-Torres, Ignacio Blanquer-Espert, Joaquín Dopazo, Ignacio Medina

**Affiliations:** ^1^Department of Computational Genomics, Centro de Investigación Príncipe Felipe (CIPF), ^2^Functional Genomics Node, (INB) at CIPF 46012, ^3^Departamento de Informática, Universidad de Valencia, 46100 Valencia, ^4^Departamento de Ingeniería y Ciencia de Computadores, Universitat Jaume I, 12071 Castellón de la Plana, ^5^Instituto de Investigación en Informática de Albacete, Universidad de Castilla-La Mancha, Campus Universitario, 02071 Albacete, ^6^Universitat Politècnica de València, Instituto de Instrumentación para Imagen Molecular, 46022 Valencia, ^7^Grupo de Investigación Biomédica de Imagen (GIBI 2^30), La Fe Polytechnic University Hospital, 46022 Valencia and ^8^Bioinformatics of Rare Diseases (BIER), CIBER de Enfermedades Raras (CIBERER), Valencia, Spain

## Abstract

HPG Aligner applies suffix arrays for DNA read mapping. This implementation produces a highly sensitive and extremely fast mapping of DNA reads that scales up almost linearly with read length. The approach presented here is faster (over 20× for long reads) and more sensitive (over 98% in a wide range of read lengths) than the current state-of-the-art mappers. HPG Aligner is not only an optimal alternative for current sequencers but also the only solution available to cope with longer reads and growing throughputs produced by forthcoming sequencing technologies.

**Availability and implementation:** https://github.com/opencb/hpg-aligner.

**Contact:** jdopazo@cipf.es or imedina@ebi.ac.uk

**Supplementary information:** Supplementary data are available at *Bioinformatics* online.

## 1 INTRODUCTION

Among the many applications of the high-throughput sequencing (HTS) technologies, DNA resequencing is probably the most extensively used because of its important clinical implications (Biesecker, 2010). The most time-consuming step in HTS data processing is the mapping process, for which many tools are already available (Fonseca *et al.*, 2012). However, while accuracy of short reads mapping process is quite reasonable, speed still remains to be an issue. And, moreover, given the way in which available mappers implement current state-of-the-art mapping algorithms, such as Burroughs-Wheeler Transform, accuracy usually falls down as read length increases because of the accumulation of errors. Therefore, there is an obvious need of new approaches that overcome these current and future problems, given that the trend in HTS technologies is to increase read length and throughput (Watson, 2014). Suffix array (SA) has recently started to be applied to accelerate DNA (Bussotti *et al.*, 2011; Chen *et al.*, 2013) or RNA (Dobin *et al.*, 2013) read mapping. Here, we propose an approach, based on SA (Mamber and Myers, 1993), that enormously increases the mapping speed without sacrificing accuracy for an ample range of read lengths.

## 2 METHODS

Our approach combines the performance of uncompressed SAs with the sensitivity of the Smith-Waterman (SW) algorithm (Smith and Waterman, 1981). SAs are data structures designed for efficient searching within a large text. Each suffix is a string starting at a certain position in the large text and ending at the end of the text. Searching within a text can be performed by binary search using the SA. Applying SA in DNA mapping, the large text is the reference genome, and the query text is the read sequence. This approach achieves an ultrafast read mapping, even in noisy scenarios with high numbers of mismatches and indels. Our mapping strategy (Fig. 1) consists of three major steps. Firstly, in the *seed searching step* the reads are split into a number of seeds distributed uniformly along the read (Supplementary Fig. S1A). HPG Aligner uses an uncompressed SA to map each of these seeds into the reference genome (Supplementary Fig. S1B). To speed up the binary search in SA, the program implements a pre-indexing strategy with a prefix table that contains all possible prefixes of length 18 (user-defined parameter). The number of all possible prefixes of length 18 exceeds by far the memory of current computers. To save memory, a compressed row storage has been implemented, where only existing prefixes are stored efficiently. For the human genome, <10 GB of memory is needed. This allows HPG Aligner to use longer prefixes to speed up searches. Secondly, in the *seed extension and clustering* step (Supplementary Fig. S1C), each mapped seed is extended in both forward and reverse directions of the read until reaching a maximum number of mismatches. Clusters of the extended seeds define the candidate alignment locations (CALs), i.e. regions that correspond to highly probable mappings of a read. CALs are formed by extended seeds within a given range of distance and longer than a threshold. Only the best CALs (that better cover the read) are selected for the next step. Finally, in the *mapping completion step*, a high-performance computing (HPC) implementation of SW attempts to fill all the gaps left in the previous step (Supplementary Fig. S1D). This implementation exploits the multiple cores of the CPUs and, within them, the Streaming SIMD Extensions (SSE) registers to achieve two levels of parallelization: (i) inter-core parallelization, by distributing batches of pairs of query sequence and reference gap sequence to be aligned among multiple cores/threads in the processor, and (ii) intra-core parallelization (Rognes and Seeberg, 2000), by processing a batch of sequence pairs using the SSE registers within a core. The use of SSE 4.2 instructions allows processing simultaneously up to four sequence alignments within each single core. Once gaps in a CAL are mapped, a score for that CAL based on user-defined penalties for mismatches and indels is calculated.

## 3 RESULTS

We have compared the proposed aligner to the most extensively used DNA-seq mappers, BWA 0.7.5a MEM (Li, 2013) and Bowtie 2 2.1.0 (Langmead and Salzberg, 2012). Benchmarks were performed in a high-end machine with two hexa-core Intel Xeon E5645 2.40 GHz CPUs and 48 GB of memory. All executions were done using the 12 cores available and memory use was monitored. HPG Aligner showed a memory peak of 32 GB.

### 3.1 Simulated data

The program dwgsim 0.1.10 from the SAMtools (http://sourceforge.net/apps/mediawiki/dnaa/index.php?title=Whole_Genome_Simulation) was used to simulate single-end reads from the human genome (Ensembl73 built upon GRCh37). The program dwgsim was run in ‘Illumina’ mode to generate datasets with 40 million reads of lengths of 100, 150, 400, 800, 2000 and 5000 bp. We generated a high-quality dataset containing 0.1% of mutations (option ‘-r 0.001’) and a second dataset with higher proportion of mutations (1% per read with option ‘-r 0.01’). In both configurations, 10% of these mutations were indels (option ‘-R 0.1’), and 30% of these indels are extended with option − X 0.30’. In addition to the mutation rate, dwgsim reproduces errors of the sequencer [-e FLOAT per base/color/flow error rate of the first read (from 0.020 to 0.020 by 0.000)]. Finally, the maximum of N’s was set to 2 (option ‘-n 2’).

Table 1 shows a comparative of HPG Aligner with BWA MEM and Bowtie 2. Reads are considered correctly mapped if chromosome, strand and position (±5bp) are coincident with the mapping coordinates, otherwise is incorrectly mapped. While percentages of correctly mapped reads were quite similar, HPG Aligner runtimes were significantly lower than BWA MEM ones, especially when read length increases, arriving to 18× for long reads (5000 bp). Bowtie 2 runtimes were even slower and the program was unable to end the mapping of reads over 800 bp. Despite other programs have been optimized for speed, like bowtie3 (Liu *et al.*, 2012) or Isaac (Raczy *et al.*, 2013), they can only deal with low error reads. Percentages of unmapped reads and incorrectly mapped reads are low for all the programs (Supplementary Table S1). The results were similar for the equivalent pair-end benchmark (Supplementary Table S2).

**Table 1.** Benchmark results comparing HPG Aligner to BWA MEM and Bowtie 2

RL	MR (%)	HPG Aligner		BWA MEM		Bowtie 2	
		CM	Time	CM	Time	CM	Time
100	0.1	98.77	20.57	96.99	29.34	94.67	29.40
	1	98.22	19.66	96.65	33.34	92.98	29.15
	2	97.45	17.46	96.11	37.62	90.52	29.04
150	0.1	99.54	22.90	98.09	43.35	96.71	47.61
	1	99.29	22.09	97.96	49.12	95.93	46.50
	2	98.96	18.13	97.72	54.03	94.73	46.36
400	0.1	99.93	31.35	99.12	124.16	98.82	209.26
	1	99.78	30.49	99.06	142.81	98.71	221.92
	2	99.58	26.30	98.95	157.65	98.56	200.11
800	0.1	99.95	35.57	99.42	279.54	99.29	4604.90
	1	99.74	35.00	99.38	312.55	99.24	2750.26
	2	99.47	34.70	99.28	340.46	99.18	2894.38

*Notes:* Percentages of correct mapping (CM) and runtimes in min (Time) are displayed for different read length in base pairs (RL) and percentages of mutation rate [MR(%)]. For RLs, >800 bp Bowtie2 was unable to finish in 3 days.

Additionally, the effect of indels was studied in other simulated datasets containing gaps of increasing size (minimum gap size of: 5, 7, 10 and 20 bp) for increasingly longer reads (100, 150, 400 and 800 bp). In the most difficult scenario (reads 800 bp long with gaps of ≥20 bp), runtimes of HPG Aligner and BWA MEM are comparable; however, HPG Aligner sensitivity is clearly higher (80.12% versus 63.73%). General performance of Bowtie 2 is comparatively poorer (Supplementary Table S3).

### 3.2 Real datasets

We have tested the aligners in a real scenario: *Drosophila* genomic sequences obtained using the PacBio technology, with ∼ 1 million long reads (Supplementary Table S4). With long reads, programs often report unrealistic alignments in which only a few tens of nucleotides were aligned while thousands were annotated as deletions. Therefore, here we consider a read correctly mapped when the mapping covers a minimum of 80% of its length. HPG Aligner was capable of mapping 93.21% of the reads, which, after removing reads below the minimal accepted covering threshold, constituted an effective 92.95% of correctly mapped reads. BWA MEM initially mapped 99.95% of the reads. However, when poorly covered reads were excluded, the effective mapping was only of 90.22%. Moreover, while HPG Aligner completed the mapping in only 27.51 min, BWA MEM required 130.34 min. This constitutes almost 5× speed-up in a real dataset, with improved alignment. Bowtie 2 could not finish the mapping, reporting systematically an out of memory error (signal 9 kill). BLASR (Chaisson and Tesler, 2012) did a good job at mapping (99.81%) but with extremely long runtime (342 min). For short read lengths, we have used a dataset of 32.7 million reads, 100 bp long, from the 1000 genomes. The relative runtimes for this length are similar to what is described in Table 1 (1.5×: HPG Aligner 14 min versus BWA MEM 21 min), as well as the mappings (96.30% HPG Aligner versus 97.13% BWA MEM, see Supplementary Table S4).

### 3.3 Other technical advantages

HPG Aligner has additional advantages. The program can directly read the FASTQ file gzipped, saving in this way both disk space and the time required for the decompression. In addition, users can specify several FASTQ files in a single command line. The mappings of each FASTQ file are concatenated in a single output file. By default, the output file is saved in the SAM format, but HPG Aligner can directly generate a BAM format file by using the option ‘--bam-format’, saving the step of SAM to BAM conversion. HPG Aligner also performs an indel realignment of mappings with the option ‘--realignment’, and a base quality score recalibration with the option ‘--recalibration’. HPC implementations of GATK recalibrator and indel realignment algorithms (McKenna *et al.*, 2010) have been included in HPG Aligner (see an example in Supplementary Fig. S2).

### 3.4 Program availability

Source code and development process has been opened to the community and released in GitHub at https://github.com/opencb/hpg-aligner. Contributions to HPG Aligner are welcome. Documentation and software are available at http://wiki.opencb.org/projects/hpg/doku.php?id=aligner:overview.

*Funding*: This work is supported by BIO2011-27069 and PRI-PIBIN-2011-1289 (Spanish Ministry of Economy and Competitiveness), the HPC4G initiative (http://www.hpc4g.org) and the Bull-CIPF Chair for Computational Genomics.

*Conflict of interest*: none declared.

## Supplementary Material

Supplementary Data
