# Supplementary material for: Acceleration of short and long DNA read mapping without loss of accuracy using suffix array
Source: Bioinformatics. 2014 Aug 20;30(23):3396–8. doi: 10.1093/bioinformatics/btu553 (PMC4816028; doi:10.1093/bioinformatics/btu553)
Supplement: Supplementary Data [file supp_30_23_3396__index.html]

Acceleration of short and long DNA read mapping without loss of accuracy using suffix array. — Acceleration of short and long DNA read mapping without loss of accuracy using suffix array — Acceleration of short and long DNA read mapping without loss of accuracy using suffix array — Supplementary Data 

# Acceleration of short and long DNA read mapping without loss of accuracy using suffix array

## Supplementary Data

files

**Files in this Data Supplement:**

- Supplementary Data - tif file
- Supplementary Data - tif file
- Supplementary Data - docx file
- Supplementary Data - docx file
- Supplementary Data - docx file
- Supplementary Data - docx file
