# Supplementary figures and images for: Acceleration of short and long DNA read mapping without loss of accuracy using suffix array
Source: Bioinformatics. 2014 Aug 20;30(23):3396–8. doi: 10.1093/bioinformatics/btu553 (PMC4816028; doi:10.1093/bioinformatics/btu553)

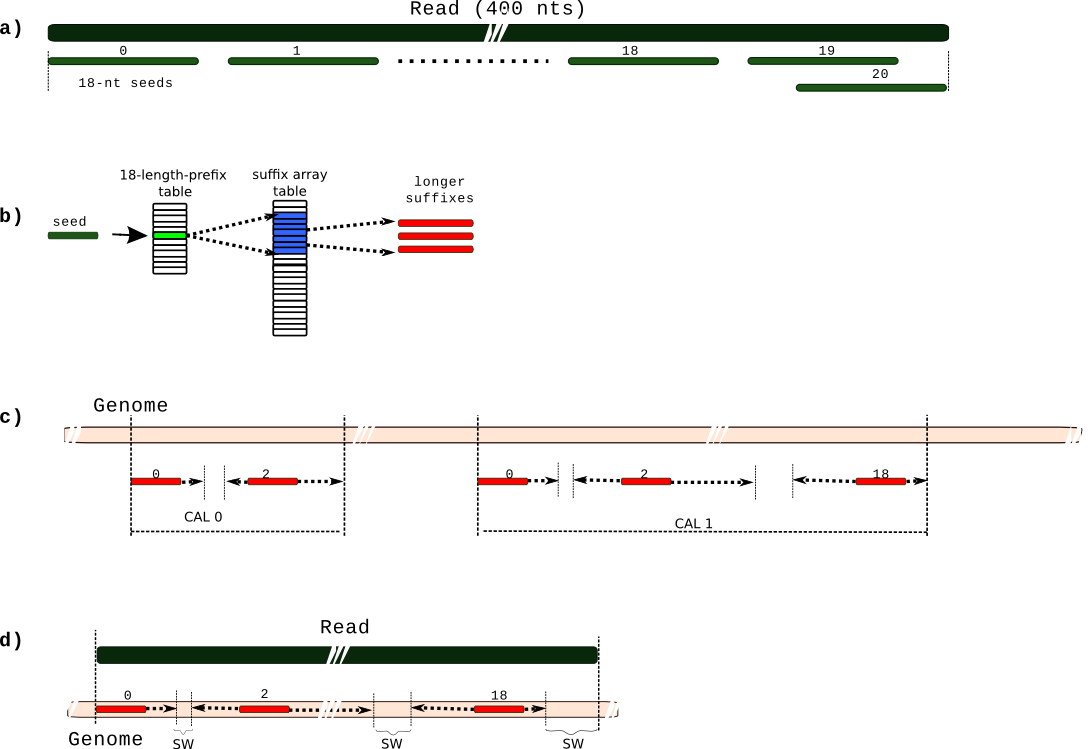

Supplement: Supplementary Data [file supp_btu553_DNA-mapper-Supplementary_Figure_1.tif]

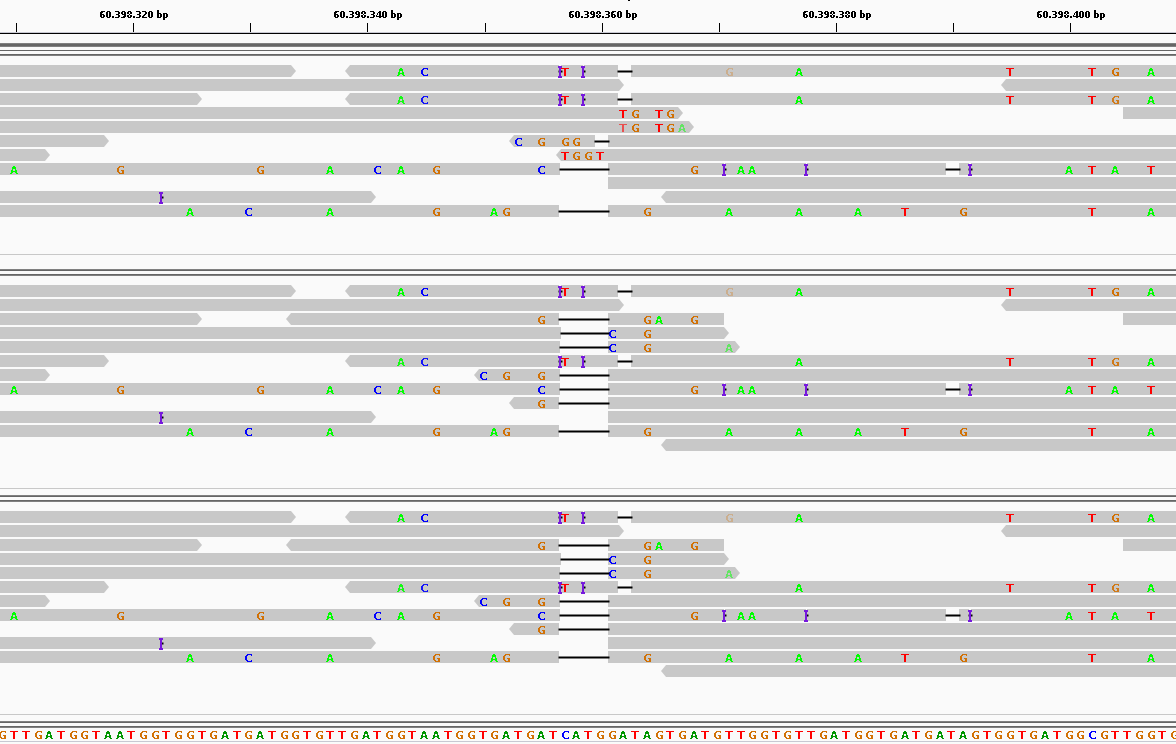

Supplement: Supplementary Data [file supp_btu553_DNA-mapper-Supplementary_Figure_2.tif]
