# Supplementary material for: Acceleration of short and long DNA read mapping without loss of accuracy using suffix array
Source: Bioinformatics. 2014 Aug 20;30(23):3396–8. doi: 10.1093/bioinformatics/btu553 (PMC4816028; doi:10.1093/bioinformatics/btu553)
Supplement: Supplementary Data [file supp_btu553_DNA-mapper-Supplementary_table_2.R1.docx]

| Supplementary Tables  Acceleration of short and long DNA read mapping without loss of accuracy using suffix array.  Joaquín Tárraga, Vicente Arnau, Héctor Martínez, Raul Moreno, Diego Cazorla, José Salavert-Torres, Ignacio Blanquer, Joaquín Dopazo and Ignacio Medina |
| --- |

^[[1]](#footnote-2)^*abstract

HPG Aligner applies suffix arrays for DNA read mapping. This implementation produces a highly sensitive and extremely fast mapping of DNA reads that scales up almost linearly with read length. The approach presented here is faster (over 20x for long reads) and more sensitive (over 98% in a wide range of read lengths) than the current, state-of-the-art mappers. HPG Aligner is not only an optimal alternative for current sequencers but also the only solution available to cope with longer reads and growing throughputs produced by forthcoming sequencing technologies.

**Supplementary Table 2**. Benchmark results with simulated pair-end reads comparing HPG Aligner to BWA MEM and Bowtie2. Percentages of reads correctly mapped (CM), reads incorrectly mapped (IM), not mapped reads (NM) and runtimes in minutes (Time) are displayed for different read length in bps (RL) and percentages of mutation rate (MR(%)). For RL 2000 and beyond Bowtie2 was unable to finish in three days.

|  |  | HPG Aligner | | | | BWA 0.7.5a MEM | | | | Bowtie2 2.1.0 | | | |
| --- | --- | --- | --- | --- | --- | --- | --- | --- | --- | --- | --- | --- | --- |
| RL | MR | CM | IM | NM | **Time** | CM | IM | NM | Time | CM | IM | NM | Time |
| 100 | 0.1% | 98.74 | 1.20 | 0.06 | 37.45 | 98.53 | 1.47 | 0.00 | 66.07 | 97.20 | 2.19 | 0.61 | 60.68 |
|  | 1% | 98.18 | 1.68 | 0.14 | 37.30 | 98.43 | 1.57 | 0.00 | 71.83 | 95.77 | 2.63 | 1.60 | 59.36 |
| 150 | 0.1% | 99.52 | 0.48 | 0.00 | 46.45 | 98.93 | 1.07 | 0.00 | 90.20 | 98.26 | 1.62 | 0.12 | 94.60 |
|  | 1% | 99.26 | 0.73 | 0.01 | 44.68 | 98.87 | 1.13 | 0.00 | 118.29 | 97.79 | 1.85 | 0.36 | 93.41 |
| 400 | 0.1% | 99.92 | 0.08 | 0.00 | 63.59 | 99.42 | 0.58 | 0.00 | 251.14 | 97.96 | 1.87 | 0.17 | 551.92 |
|  | 1% | 99.74 | 0.26 | 0.00 | 61.64 | 99.37 | 0.63 | 0.00 | 595.58 | 97.69 | 2.02 | 0.29 | 479.41 |
| 800 | 0.1% | 99.94 | 0.06 | 0.00 | 74.89 | 99.62 | 0.38 | 0.00 | 559.72 | 98.75 | 1.20 | 0.05 | 4,553.98 |
|  | 1% | 99.73 | 0.26 | 0.00 | 72.17 | 99.58 | 0.42 | 0.00 | 631.76 | 98.61 | 1.32 | 0.07 | 4,689.93 |
| 2000 | 0.1% | 99.94 | 0.06 | 0.00 | 123.45 | 99.81 | 0.19 | 0.00 | 1,546.40 | - | - | - | - |
|  | 1% | 99.67 | 0.33 | 0.01 | 128.40 | 99.77 | 0.23 | 0.00 | 1,609.49 | - | - | - | - |
| 5000 | 0.1% | 99.91 | 0.09 | 0.00 | 255.54 | 99.73 | 0.27 | 0.00 | 5,121.77 | - | - | - | - |
|  | 1% | 99.67 | 0.29 | 0.00 | 266.65 | 99.59 | 0.41 | 0.00 | 5,337.43 | - | - | - | - |

1. *To whom correspondence should be addressed. [↑](#footnote-ref-2)
