# Supplementary material for: Acceleration of short and long DNA read mapping without loss of accuracy using suffix array
Source: Bioinformatics. 2014 Aug 20;30(23):3396–8. doi: 10.1093/bioinformatics/btu553 (PMC4816028; doi:10.1093/bioinformatics/btu553)
Supplement: Supplementary Data [file supp_btu553_DNA-mapper-Supplementary_table_4.R1.docx]

| Supplementary Tables  Acceleration of short and long DNA read mapping without loss of accuracy using suffix array.  Joaquín Tárraga, Vicente Arnau, Héctor Martínez, Raul Moreno, Diego Cazorla, José Salavert-Torres, Ignacio Blanquer, Joaquín Dopazo and Ignacio Medina |
| --- |

^[[1]](#footnote-2)^*abstract

HPG Aligner applies suffix arrays for DNA read mapping. This implementation produces a highly sensitive and extremely fast mapping of DNA reads that scales up almost linearly with read length. The approach presented here is faster (over 20x for long reads) and more sensitive (over 98% in a wide range of read lengths) than the current, state-of-the-art mappers. HPG Aligner is not only an optimal alternative for current sequencers but also the only solution available to cope with longer reads and growing throughputs produced by forthcoming sequencing technologies.

**Supplementary Table 4**. Mapping of reads from real datasets. PacBio reads consist of about 1 million of *Drosophila* genomic sequences with an average read length 8,697 (1 021 564 reads, mean length 8697bp, median length 7750bp, longest read of. 36765 bp; available at: ftp://cbcb.umd.edu/pub/data/sergek/dros_corrected.fastq.bz2). The reads were corrected at accuracy over 99.3%, according to the submitters). The reads were mapped against the *Drosophila* *melanogaster* reference genome (assembly BDGP5.25, Ensembl release 64). Short reads consist of 32.7 million reads 100bp long; from the 1000 genomes repository (file SRR642644_1.filt.fastq.gz, available at ftp://ftp.1000genomes.ebi.ac.uk/vol1/ftp/data/150189/sequence_read/). The reads were mapped against the human genome (Ensembl73 built upon GRCh37). Percentages of reads mapped (RM), reads correctly mapped with alignment covering more than 80% of the length of the read only computed in the case of long reads (CM), and runtimes in minutes (Time) are displayed for the two real datasets above mentioned with different read length bps (RL) and sizes, expressed in million reads (NR).

|  |  |  | **HPG Aligner** | | | **BLASR** | | | **BWA 0.7.5a MEM** | | | **Bowtie2 2.1.0** | | |
| --- | --- | --- | --- | --- | --- | --- | --- | --- | --- | --- | --- | --- | --- | --- |
|  | **NR** | **RL** | **RM** | **CM** | **Time** | **RM** | **CM** | **Time** | **RM** | **CM** | **Time** | **RM** | **CM** | **Time** |
| *Drosophila* PacBio dataset | 1.02 | 8,697 | 93.21 | 92.95 | 27.51 | 99.81 | 99.81 | 342.00 | 99.95 | 90.09 | 130.34 | NA* | NA* | NA* |
| human, SRR642644_1.filt, 34.7 M, 100nt | 34.70 | 100 | 96.30 | 96.30 | 14.70 | 46.01 | 46.01 | 212.46 | 97.13 | 97.13 | 21.02 | 64.48 | -- | 24.35 |

* Bowtie2-aligned died with signal 9 (KILL) in a running environment with 48GB of RAM memory

1. *To whom correspondence should be addressed. [↑](#footnote-ref-2)
